# Application of alpha1-antitrypsin in a rat model of veno-arterial extracorporeal membrane oxygenation

**DOI:** 10.1038/s41598-021-95119-y

**Published:** 2021-08-04

**Authors:** Fabian Edinger, Christoph Schmitt, Christian Koch, J. Michael McIntosh, Sabina Janciauskiene, Melanie Markmann, Michael Sander, Winfried Padberg, Veronika Grau

**Affiliations:** 1 Department of Anesthesiology, Intensive Care Medicine and Pain Therapy, Justus-Liebig University of Giessen, Giessen, Germany; 2 George E. Wahlen Veterans Affairs Medical Center, Salt Lake City, UT USA; 3 Department of Biology, University of Utah, Salt Lake City, UT USA; 4 Department of Psychiatry, University of Utah, Salt Lake City, UT USA; 5 Department of Respiratory Medicine, Hannover Medical School, German Centre for Lung Research (DZL), Hannover, Germany; 6 Laboratory of Experimental Surgery, Department of General and Thoracic Surgery, German Centre for Lung Research (DZL), Justus-Liebig-University of Giessen, Giessen, Germany

**Keywords:** Cardiac device therapy, Acute inflammation, Translational research

## Abstract

Extracorporeal membrane oxygenation (ECMO) is a life-saving intervention for patients suffering from respiratory or cardiac failure. The ECMO-associated morbidity and mortality depends to a large extent on the underlying disease and is often related to systemic inflammation, consecutive immune paralysis and sepsis. Here we tested the hypothesis that human α1-antitrypsin (SERPINA1) due to its anti-protease and anti-inflammatory functions may attenuate ECMO-induced inflammation. We specifically aimed to test whether intravenous treatment with α1-antitrypsin reduces the release of cytokines in response to 2 h of experimental ECMO. Adult rats were intravenously infused with α1-antitrypsin immediately before starting veno-arterial ECMO. We measured selected pro- and anti-inflammatory cytokines and found, that systemic levels of tumor necrosis factor-α, interleukin-6 and interleukin-10 increase during experimental ECMO. As tachycardia and hypertension developed in response to α1-antitrypsin, a single additional bolus of fentanyl and midazolam was given. Treatment with α1-antitrypsin and higher sedative doses reduced all cytokine levels investigated. We suggest that α1-antitrypsin might have the potential to protect against both ECMO-induced systemic inflammation and immune paralysis. More studies are needed to corroborate our findings, to clarify the mechanisms by which α1-antitrypsin inhibits cytokine release in vivo and to explore the potential application of α1-antitrypsin in clinical ECMO.

## Introduction

Extracorporeal membrane oxygenation (ECMO) is a life-saving intervention for patients suffering from respiratory or cardiac failure. It is mainly used as a bridge to recovery, to implantation of artificial hearts and to pulmonary or cardiac transplantation. Depending on the underlying disease veno-venous ECMO (VV ECMO) or veno-arterial ECMO (VA ECMO) is used. While VV ECMO is suitable for patients suffering from respiratory failure, VA ECMO is indicated for patients with cardiocirculatory insufficiency. The number of centers offering ECMO and the number of treated patients increased strongly during the last decade (www.elso.org)^1^. However, the ECMO-associated morbidity and mortality is dependent on the experience of the center and of the underlying indication. Compared to a 60-day mortality of 34% for patients with acute respiratory distress syndrome and VV ECMO, hospital discharge of 30.8% is reported for patients treated with VA ECMO due to postcardiotomy cardiogenic shock^2,3^. Intracerebral hemorrhage^4^ and systemic inflammation^5–7^ significantly contribute to the ECMO-associated mortality.

ECMO inevitably involves the contact of blood with foreign surfaces, like the membrane and circuit tubing, as well as mechanical damage of blood cells by pumps and increased shear stress. Mechanical cell damage results in spillage of cytoplasm that contains ATP as well as numerous other danger-associated molecular patterns (DAMPs), known activators of innate immunity. Within the first few hours, ECMO induces a multitude of intricate and still not fully understood pro-inflammatory mechanisms including activation of endothelial cells, neutrophils, monocytes, mast cells, and platelets as well as the induction of complement and coagulation cascades, the production of reactive oxygen species, and the release of pro- and anti-inflammatory cytokines such as tumor necrosis factor-α (TNF-α), interleukin (IL)-1β, IL-6 and IL-10^5,7,8^. Consequently, ECMO often leads to the development of an initially sterile systemic inflammatory response syndrome (SIRS) that paves the way to life-threatening immune paralysis and multi organ damage. SIRS can promote the development of acute respiratory distress syndrome and sepsis due to protease- and cytokine-induced barrier dysfunction of endothelia and epithelia of the respiratory and gastrointestinal system^5,7–10^.

In order to investigate VA ECMO and its hemodynamic as well as inflammatory implications, several models of extracorporeal circulation have already been established in the rat^11–18^. However, depending on the underlying scientific question, the experimental methods vary broadly. Most animal models simulate a cardiopulmonary bypass, without perfusion of the lungs, or a deep hypothermic cardiac arrest with extracorporeal reperfusion. Accordingly, these models are designed to study ischemia/reperfusion injury along with an extracorporeal circuit for gas exchange. Also, the cannulation strategies vary. Most of these models use the right internal jugular vein for the inflow of venous blood to the ECMO (venous drainage) and the tail artery for the arterial outflow from the ECMO back to the circulation (arterial return). The use of the femoral artery or the carotid artery for returning the oxygenized and carbon dioxide cleared blood to the circulation result in different hemodynamic situations^17,19,20^. Our approach is based on the experimental model described by Koning et al. with retrograde arterial return via the right femoral artery^17^. In contrast to Koning et al., we continue invasive ventilation of the lungs during VA ECMO to avoid pulmonary hypoxia, because we aim to investigate the inflammatory response induced by the ECMO circuit^21^.

Preparations of the serine proteinase inhibitor α1-antitrypsin (AAT, SERPINA1), that is used clinically for the treatment of AAT-deficient patients^22,23^, has the potential to attenuate most of the above described pathogenic mechanisms^24^. The capacity of AAT to antagonize numerous proteases including trypsin, neutrophil elastase, protein 3, and mast cell tryptase, is investigated in detail. In addition, AAT impairs the degranulation of neutrophils and inactivates reactive oxygen species^25–28^. However, AAT is functionally inactivated, when interacting with proteases or reactive oxygen species^27,29,30^, which may eventually result in acquired functional AAT deficiency in patients undergoing ECMO.

Increasing clinical and experimental evidence suggests potent anti-inflammatory functions of AAT beyond its anti-protease function^30–32^. AAT-deficient patients are prone to develop inflammatory diseases such as vasculitis, panniculitis fibromyalgia or bronchial asthma. Augmentation therapy with AAT preparations was applied in numerous cases with tremendous success^30^. Further, AAT protects from inflammatory diseases in experimental animals including ischemia/reperfusion injury, islet allograft rejection, graft-versus-host-disease, and rheumatoid arthritis^33–36^. The pro-inflammatory effects of lipopolysaccharide, a cell wall constituent of Gram-negative bacteria, are attenuated by AAT, and even anti-bacterial functions of AAT have been reported^37,38^. In addition, AAT has been shown to regulate chemotaxis and cell adhesion by reducing the expression of chemokines^27,39–41^. In the same line, the gene expression of Toll-like receptor 4, IL-1β and TNF-α is down-modulated, and the expression anti-inflammatory mediators IL-10 and IL-1 receptor antagonist are up-regulated^42–44^. Recently, we discovered an efficient AAT-induced reduction of ATP-induced secretion of IL-1β by monocytes and lung tissue, by a mechanism that involves nicotinic acetylcholine receptors (nAChRs)^45^.

In this explorative study, we test the hypothesis that intravenous treatment with AAT reduces the release of cytokines in response to VA ECMO.

## Results

### Characteristics of experimental ECMO

**Figure 1:**
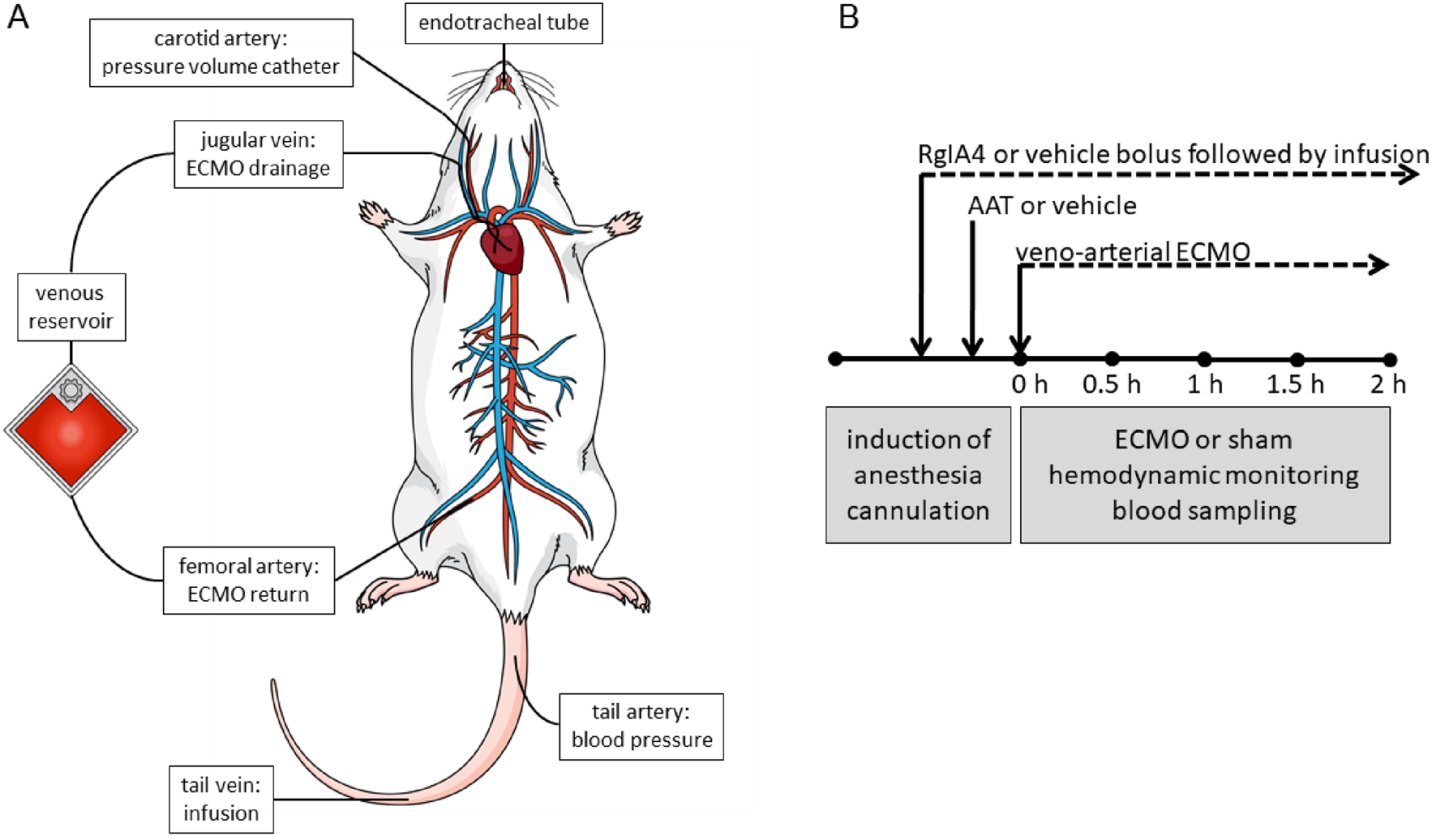
Design of the study. (**A**) Cannulation strategy used for experimental veno-arterial extracorporeal membrane oxygenation (ECMO) in the rat. The animals were endotracheally intubated and ventilated. The right atrium was cannulated via the jugular vein for venous blood drainage. Venous blood passed through the ECMO device and the oxygenized and carbon dioxide cleared blood was returned via the femoral artery. A pressure volume catheter was placed into the left ventricle through the right carotid artery. The peripheral blood pressure was measured in the tail artery. The lateral tail vein was punctured for infusion of medicaments and the test substances under investigation. (**B**) Graphical visualization of the time course of the experiments. Rats were anesthetized and treated with the conopeptide RgIA4 or vehicle, which were given as a bolus followed by continuous infusion. α1-antitrypsin (AAT) or vehicle were applied by syringe pump over 5 min. Thereafter, veno-arterial ECMO was started and hemodynamic monitoring as well as blood sampling was done for 2 h. Thereafter, animals were sacrificed. The same procedure was applied to sham-treated animals, but the ECMO was omitted.

**Figure 2:**
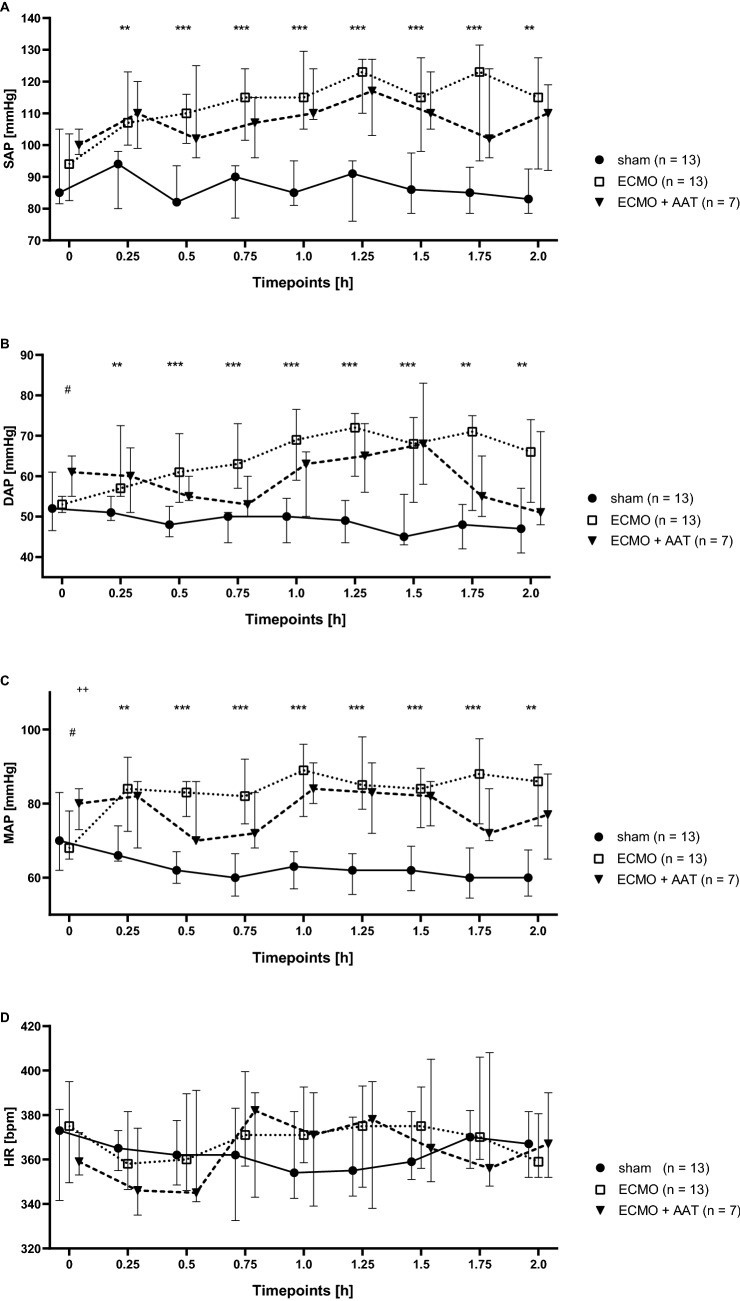
Arterial blood pressure and heart rate (HR) in experimental rats. Animals underwent the sham procedure (n = 13), extracorporeal membrane oxygenation (ECMO, n = 13) or ECMO combined with application of α1-antitrypsin (AAT, n = 7). Values were recorded every 15 min until the end of the experiments. The systolic arterial blood pressure (SAP, (**A**)), the diastolic arterial blood pressure (DAP, (**B**)) and the mean arterial blood pressure (MAP, (**C**)) were significantly elevated in animals connected to ECMO compared to the sham group. These parameters were not changed in rats treated with AAT. The HR did not significantly differ among all groups (**D**). Data are presented as median and interquartile ranges 25% and 75%; Kruskal–Wallis test followed by pairwise Wilcoxon–Mann–Whitney test; **p ≤ 0.01, ***p ≤ 0.001 sham versus ECMO; ^#^*p* ≤ 0.05, ECMO versus ECMO + AAT.

**Figure 3:**
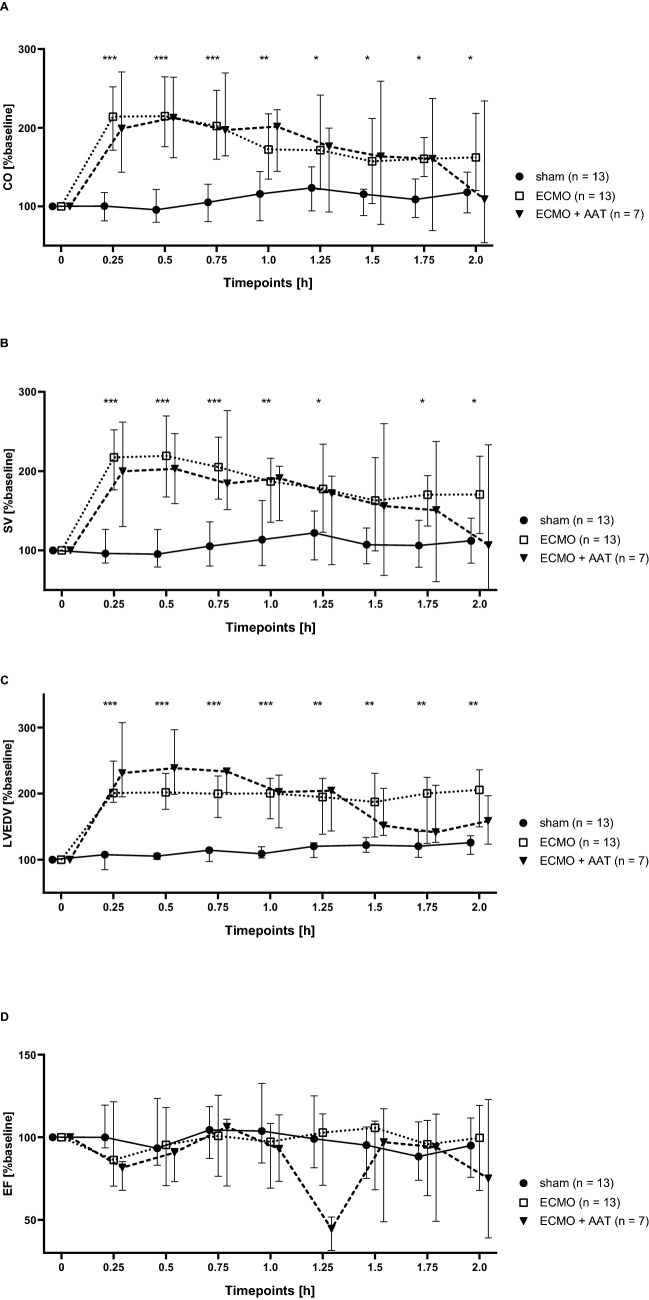
Hemodynamic parameters in experimental rats. Animals underwent the sham procedure (n = 13), extracorporeal membrane oxygenation (ECMO, n = 13) or ECMO combined with application of α1-antitrypsin (AAT, n = 7). (**A**) We found an increased cardiac output (CO) in response to ECMO compared to the sham group, irrespective of treatment with AAT. Similar results were found regarding stroke volume (SV, (**B**)) and left ventricular end-diastolic volume (LVEDV, (**C**)). (**D**) The ejection fraction (EF) was similar among most experimental groups and time points investigated. Only 75 min after starting ECMO, the EF was reduced in ECMO + AAT compared to ECMO alone. Values were recorded every 15 min until the end of the experiments. Data are presented as median and interquartile ranges 25% and 75%; Kruskal–Wallis test followed by pairwise Wilcoxon–Mann–Whitney test; *p ≤ 0.05, **p ≤ 0.01; ***p ≤ 0.001 sham versus ECMO.

The cannulation strategy used in this study for experimental VA ECMO in the rat is visualized in Fig. 1A and a graphical summary of the time course of the experiments is given in Fig. 1B. After commencing VA ECMO, hemodynamic variables were recorded every 15 min until the end of the experiment. The systolic, diastolic and mean arterial blood pressures (SAP, DAP, MAP) were significantly elevated in animals connected to VA ECMO compared to the sham group (Fig. 2A–C). Apart from DAP and MAP baseline values, no statistically significant differences in SAP, DAP and MAP were measured between the VA ECMO group and animals connected to VA ECMO and treated with AAT (ECMO + AAT) (Fig. 2A–C). The heart rate (HR) did not significantly differ among all groups (Fig. 2D). Until the end of the experiment, we found an increased native cardiac output (CO) in animals treated with VA ECMO compared to the sham group, irrespective of treatment with AAT (Fig. 3A). Similar results were found regarding stroke volume (SV; Fig. 3B) and left ventricular end-diastolic volume (LVEDV; Fig. 3C). The left ventricular ejection fraction (EF) was similar among most experimental groups and time points investigated (Fig. 3D).

Hemoglobin, blood gas, glucose and lactate data are presented in Table 1. Besides a significant decrease in hemoglobin concentrations after commencing VA ECMO, a significant increase in lactate was measured after 2 h of VA ECMO compared to the sham group. Further, a significantly elevated oxygenation and carbon dioxide clearance was seen after starting VA ECMO compared to sham, and a significantly decreased pH was measured in the sham group in comparison to the ECMO group (Supplementary Table S1). No dramatic changes in electrolytes were observed (Supplementary Table S1).

**Table 1:** Blood parameters.

Parameter	Group	0 h	1 h	2 h
Hb [g/dL]	Sham	13.4 (13.0–14.1)	12.3 (11.8–13.3)	11.3 (10.6–11.9)
	ECMO	14.0 (13.6–14.4)*	6.9 (6.6–7.3)***	6.6 (5.9–7.1)***
	ECMO + AAT	13.8 (12.4–14.3)	6.9 (6.3–7.1)	6.8 (6.3–7.4)
	ECMO + AAT + RgIA4	13.1 (12.0–13.6)	6.6 (6.5–7.0)	6.7 (6.6–6.9)
pO_2_ [mm Hg]	Sham	166 (135–183)	138 (120–164)	126 (106–144)
	ECMO	168 (148–195)	236 (202–261)***	240 (197–256)***
	ECMO + AAT	162 (155–193)	223 (215–232)	218 (170–225)
	ECMO + AAT + RgIA4	174 (167–186)	228 (221–238)	202 (184–218)
pCO_2_ [mm Hg]	Sham	38 (37–46)	42 (38–48)	43 (41–48)
	ECMO	41 (39–45)	32 (30–37)**	35 (32–42)*
	ECMO + AAT	40 (35–40)	36 (35–38)	36 (34–42)
	ECMO + AAT + RgIA4	40 (35–41)	32 (32–36)	36 (33–37)
Glucose [mg/dL]	Sham	103 (96–120)	99 (96–125)	103 (92–114)
	ECMO	116 (111–123)	125 (116–134)*	109 (103–115)
	ECMO + AAT	113 (95–133)	126 (119–135)	118 (98–122)
	ECMO + AAT + RgIA4	106 (99–123)	111 (107–113)	116 (105–117)
Lactate [mmol/L]	Sham	0.9 (0.9–1.4)	0.9 (0.8–1.3)	0.8 (0.7–0.9)
	ECMO	1.0 (0.9–1.1)	1.1 (0.8–1.5)	1.3 (1.0–3.2)**
	ECMO + AAT	0.9 (0.8–1.0)	1.1 (0.8–1.3)	1.5 (0.9–2.6)
	ECMO + AAT + RgIA4	1.1 (0.9–1.1)	0.8 (0.8–0.9)	1.1 (1.0–1.1)

Data are presented as median and interquartile ranges (25th and 75th percentile); Kruskal–Wallis test followed by pairwise Wilcoxon–Mann–Whitney test.

*AAT* α-1-antitrypsin, *ECMO* extracorporeal membrane oxygenation, *Hb* haemoglobin, *pO*_*2*_ oxygen partial pressure, *pCO*_*2*_ carbon-dioxide partial pressure.

*p ≤ 0.05; **p ≤ 0.01; ***p ≤ 0.001; *sham vs. ECMO.

### Systemic cytokine levels during ECMO

**Figure 4:**
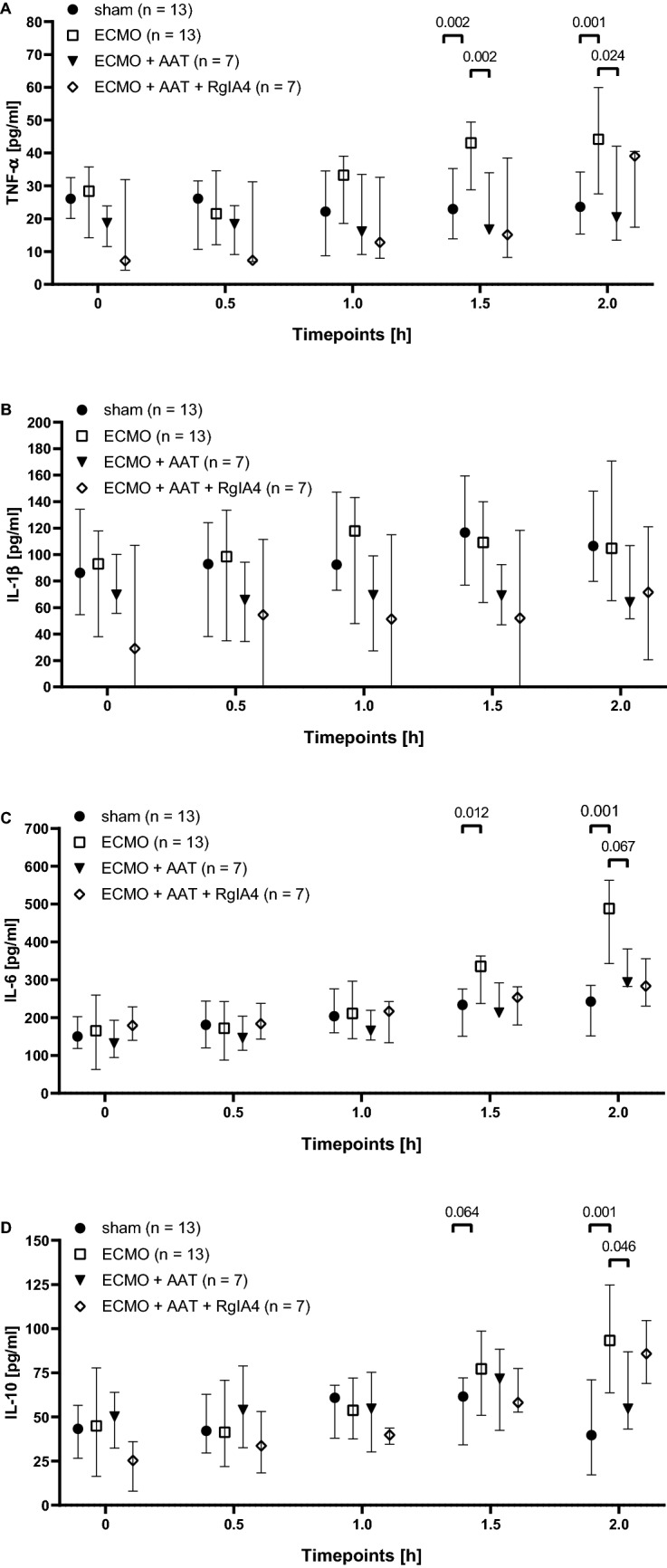
Cytokine concentrations in experimental rats. Animals underwent the sham procedure (n = 13), extracorporeal membrane oxygenation (ECMO, n = 13) ECMO combined with application of α1-antitrypsin (AAT, n = 7) or ECMO combined with AAT and the conopeptide RgIA4 (n = 7). (**A**) Tumor necrosis factor-α (TNF-α) levels increased after 1.5 and 2 h of ECMO compared to sham, treatment with AAT reduced this increase. The effect of AAT was not sensitive to RgIA4. (**B**) No significant changes in circulating interleukin (IL)-1β levels were seen in none of the experimental groups. IL-6 (**C**) and IL-10 (**D**) levels increased after 1.5 and 2 h of ECMO compared to sham and treatment with AAT reduced their increase 2 h after starting ECMO. Again, RgIA4 did not cause any changes (**C,D**). Cytokines were measured every 30 min until the end of the experiments. Data are presented as median and interquartile ranges 25% and 75%; Kruskal–Wallis test followed by pairwise Wilcoxon-Mann–Whitney test; p-values are indicated in the graphs.

Blood was drawn before starting VA ECMO and at 30 min intervals thereafter (n = 13). Cytokine levels were measured in the blood plasma from rats undergoing VA ECMO and compared to sham-operated animals (n = 13, each). IL-6 (*p* = 0.012) and TNF-α (*p* = 0.002) levels significantly increased after 90 min of VA ECMO and IL-10 tended to be elevated (*p* = 0.064), but no increase was seen for IL-1β (*p* = 0.266; Fig. 4A–D). After another 30 min of VA ECMO, IL-6 (*p* = 0.001), TNF-α (*p* = 0.001) and IL-10 levels (*p* = 0.001) were significantly increased (Fig. 4B–D). Again, no significant differences were seen between sham- and VA ECMO-treated rats in the blood levels of IL-1β, 2 h after starting VA ECMO (Fig. 4A).

### Effect of AAT on cytokine levels

Plasma levels of TNF-α were significantly reduced after 90 min (*p* = 0.002) and 2 h of VA ECMO (*p* = 0.024) in the presence of AAT (100 mg/kg body weight; n = 7) and increased sedative doses. In addition, a tendency towards reduced concentrations of IL-6 (*p* = 0.067) and significantly reduced levels of IL-10 (*p* = 0.046) were measured after 2 h of VA ECMO. AAT did not change the plasma levels of IL-1β (Fig. 4A–D). In all experimental groups in which AAT was given, acute onset of tachycardia and hypertension was observed after about 1 h of VA ECMO, and the animals received a single bolus of fentanyl 6 µg/kg body weight and midazolam 1.5 mg/kg body weight.

### Effects of the nAChR antagonist RgIA4

We combined the conopeptide RgIA4, a specific antagonist of nAChRs containing subunits α9/α10^46^, with AAT in ECMO-treated rats to test the hypothesis, that these receptors are involved in the reduction of cytokine levels^45^. No significant difference was seen, when comparing the experimental groups ECMO + AAT to ECMO + AAT + RgIA4.

## Discussion

The results of this explorative study can be summarized as follows: (i) the experimental model of VA ECMO in the rat causes characteristic hemodynamic changes; (ii) experimental VA ECMO induces inflammation that is reflected by increased levels of circulating IL-6 and TNF-α; (iii) treatment with AAT and higher sedation reduces the levels of these cytokines. These data are in line with our hypothesis that AAT exerts anti-inflammatory effects in the context of VA ECMO. However, due to the limitations discussed below, more research in warranted to confirm or refuse this hypothesis.

While the venous drainage of VA ECMO is most often realized via the right atrium, the arterial return differs. Due to its lower risk, the femoral artery is frequently cannulated percutaneously for blood return from the extracorporeal circulation^47^. We adopted this approach, because the alternative central cannulation that requires an open thorax causes more surgery-associated inflammation, which might obscure the inflammation caused by the extracorporeal circuit and VA ECMO. Further, to reduce flow speed and hemolysis, the cannula with the biggest diameter (1.7 mm) was used, which could be inserted into the jugular vein. Since the measurement of markers of hemolysis would require additional blood withdrawal and thus enhance dilutional anemia, they were not performed. Nevertheless, it has to be noted that hemolysis leads to vasoconstriction and activation of the coagulation system.

The observed elevation of the blood pressure after commencing the ECMO is in accordance with previous studies in rats with femoral VA ECMO^13,15,17^. Since the HR did not differ between the groups, the elevated CO is caused by an increased SV. It should be taken into account, that the femoral ECMO return leads to a retrograde perfusion with a watershed in the aortic arch^48,49^. Since the LVEDV was elevated during VA ECMO, the watershed increased the left ventricular afterload in our study. Interestingly, the increased SV and consecutive CO seems to be caused by the ECMO. After commencing the ECMO a right shift of the pressure volume loops could be observed, which indicates an increased stroke work. We should be aware, that healthy rats were treated with a femoral VA ECMO. Contrary to cardiopulmonary bypass with cardiac arrest, the heart was continuously beating during VA ECMO and the CO could be measured with a pressure–volume catheter. Animals of the ECMO + AAT group showed signs of decreased anesthetic effects like tachycardia with hypertension after about 1 h of VA ECMO and, therefore, the anesthesia was adapted with a bolus of fentanyl 6 µg/kg bodyweight and midazolam 1.5 mg/kg bodyweight. Hence, the observed decrease of the EF could be caused be the negative inotropic effects of fentanyl, midazolam and isoflurane. Maybe, AAT interacted with the anesthetics and slightly impaired their function.

The decline in hemoglobin in the ECMO group can be explained by hemodilution, which is induced by the priming volume of 12 mL. These results are similar to published results of rat ECMOs using the same oxygenator^14,17,19^. Further, the reduced levels of hemoglobin and elevated concentrations of lactate after 2 h of VA ECMO might indicate an insufficient oxygen supply. Following this assumption, a reduction of the priming volume by shortening the circuit tubing in combination with the same oxygenator resulted in higher hemoglobin values and no increase in lactate values was observed in this setting within 2 h of VA ECMO^21^. The increased oxygenation and carbon dioxide clearance during VA ECMO can be explained by the continuous ventilation of the experimental animals. Koning et al. administered CO_2_ to the ECMO gas flow^17^, while in our approach the paCO_2_ could be regulated only by the gas flow on the membrane without additional CO_2_.

Numerous publications indicate that the mainly pro-inflammatory cytokines IL-1β, IL-6, IL-8 and TNF-α play important roles in the inflammatory response to ECMO or cardiopulmonary bypass^7,8,50^. In addition, the anti-inflammatory cytokine IL-10 is of interest because of its presumably protective functions regarding sterile SIRS but also as an indicator of a compensatory anti-inflammatory response syndrome that impairs host defense against infections^7^. As the gene for IL-8 is absent from rodents, the expression of this cytokine cannot be investigated in the rat. Hence, we decided to investigate circulating levels of IL-1β, IL-6, IL-10, and TNF-α in our study.

Our data might suggest that IL-1β does not play an important role in our experimental model for VA ECMO, as we did not observe an increase in IL-1β levels during VA ECMO compared to sham. In the same line, increased levels of circulating IL-1β were first measured after 3 h of ECMO in piglets^8^. The synthesis and release of IL-1β are tightly controlled and typically require two successive danger signals^51–54^. In the context of ECMO the first signal may be DAMPs released from damaged cells or bacterial cell wall components that induce the biosynthesis of pro-IL-1β. Extracellular ATP originating from the cytoplasm of damaged or lysed cells is a typical second signal that induces inflammasome assembly, activation of caspase-1, cleavage of pro-IL-1β and the release of mature bioactive IL-1β^51–54^. Hence, it is conceivable that IL-1β is not released within the first 2 h of VA ECMO, when initially healthy animals are investigated. Alternatively, the extremely short half-live of IL-1β in the circulation, might have prevented the detection of VA ECMO-induced changes^55,56^. If this holds true, circulating IL-6 and TNF-α levels might reflect preceding changes in IL-1β, as these cytokines can be induced by IL-1β.

We observed a modest but significant increase in IL-6 levels in response to 90 min or 2 h of ECMO. A similar although not significant increase was described for piglets^8^. High circulating levels of IL-6 were identified as a poor prognostic factor in a small study on human ECMO including pediatric and adult patients^50^. IL-6 is predominantly produced by mononuclear phagocytes and by epithelial cells in response to stimulation of Toll-like receptors, IL-1 or TNF-α signaling and is directly released upon biosynthesis^57,58^. Although circulating IL-6 is without a doubt a marker for ongoing inflammation, its function is less clear-cut as it induces further pro-inflammatory mechanisms but also negative feed-back loops that regulate inflammation including the upregulation of the cytokine IL-10^57,58^.

The levels of circulating IL-10 tended to be increased 90 min after starting VA ECMO and were significantly increased after 2 h. IL-10 is well-known for its anti-inflammatory functions and is expressed together with pro-inflammatory cytokines by virtually all leukocytes in response to multiple agonists of pattern recognition receptors as well as to several cytokines. There are, however, new insights on additional pro-inflammatory roles of IL-10 promoting effector cell survival and proliferation, as well as on its regulation by metabolic switches^59^. Interestingly, an accumulation of lactate in areas of reduced perfusion induces the expression of IL-10^59^. The precise role of IL-10 in ECMO-associated systemic inflammation needs to be investigated in detail. It is most likely protective but might also impair host defense and increase the risk of infectious complications.

Finally, we observed a clear increase in circulating TNF-α in VA ECMO-treated rats compared to sham. A similar rise in TNF-α levels has been observed in piglets within the first h of ECMO^8^ and TNF-α was also shown to increase in response to human ECMO^7,50^. TNF-α is predominantly produced by monocytes/macrophages and, to a lesser extent, by other cells such as lymphocytes, mast cells or fibroblasts^60^. It is synthesized as a transmembrane protein that can be cleaved to its soluble form by the TNF-converting metallopeptidase domain 17 (ADAM17)^60^. Most interestingly, TNF-α was shown to be swiftly released by intestinal mast cells during ECMO in piglets^8^. This peculiar mechanism is of interest, because intestinal damage seems to play a pivotal role in ECMO-induced systemic inflammation^7,10^. TNF-α is a potent pro-inflammatory cytokine that exerts pleiotropic functions ranging from an activation of innate immune cells to the induction of cell death^60^.

Most of the reported pro-inflammatory and anti-inflammatory cytokine levels of other experimental settings in the rat with an extracorporeal circulation are higher than our results. However, these models include hypoxic cardiac arrest and deep hypothermic arrest^14,15,18,19^. Most likely ischemia/reperfusion injury, which is avoided in our experimental setting, importantly increased systemic inflammation in these studies. Nevertheless, it must be mentioned that the use of a venous reservoir in our approach could enhance the inflammation in all ECMO groups. The reservoir was used to avoid roller pump-associated negative pressure related gas release during inadequate venous drainage.

AAT was applied i.v. before ECMO to test the hypothesis, that AAT blunts VA ECMO-induced systemic inflammation. Assuming a blood volume of 85 mL/kg body weight, an injection of 100 mg/kg AAT should initially result in a concentration of 1.2 mg/mL, which is within the physiological range in humans^61^. As AAT has a half-life of 3 to 5 days in humans^61^, we applied AAT as a bolus and expect that sufficient AAT was present in the circulation of the rat during the experiment. AAT infusion reduced IL-6 and TNF-α levels measured at the end of the experiment. An AAT-mediated reduction of the expression of these pro-inflammatory cytokines during ECMO is expected, because numerous publications document similar anti-inflammatory effects in the presence of pro-inflammatory stimuli, both in in vitro and in vivo^27,34–36,38,41,62–66^. There are, however, also conflicting data suggesting that in the short-term similar to our experiments, AAT enhances the lipopolysaccharide-induced release of TNF-α and IL-6 by primary human monocytes^64^. However, the reduction in circulating IL-10 levels is surprising, as AAT was shown to increase the release of this anti-inflammatory cytokine during inflammation^42–44,65,67^. Our conflicting results might be explained by differences in the experimental settings including different time courses used in previous studies.

Mechanistically, AAT has the potential to inhibit ECMO-induced inflammation at different levels. It may for example reduce the above-described protease-induced barrier dysfunction of the gastrointestinal system and, hence reduce the translocation of bacterial pyrogens into the circulation. The reduction of circulating IL-6 and TNF-α can be further explained by an interference of AAT with the NF-κB system, which would result in reduced mRNA expression of both cytokines^68^. In comparison to IL-6, the effects of AAT on TNF-α were seen earlier during the experiment and seemed to be more robust. Primarily, TNF-α is a membrane-spanning cell surface protein, that can be released into the circulation upon cleavage by the protease ADAM17. Although ADAM17 is not a serine protease, AAT has been shown to bind to ADAM17 and to inhibit its function^39,66,69^, which might also play a role in our experiments. We demonstrated before, that the nAChR subunit α9 plays an essential role in the AAT-mediated inhibition of ATP-induced inflammasome activation and IL-1β release by human monocytes^45^. To test the hypothesis that this mechanism directly or indirectly contributes to the anti-inflammatory effects of AAT in experimental ECMO, the α9/α10-specific conopeptide RgIA4^46^ was applied along with AAT. We injected a bolus of 2 nmol RgIA4 per rat, which should result in a concentration of about 6 nmol/L in the total body. This concentration was shown to antagonize nAChRs containing subunits α9 and α10^46^. In the light of a half-life below 20 min in rodents, the bolus injection of RgIA4 was followed by a continuous infusion of 38.8 nmol RgIA4 per kg and h, to keep the concentration of RgIA in the range of 6 nmol/L throughout the experiment. However, no significant differences in cytokine levels were seen between ECMO + AAT and ECMO + AAT + RgIA4, which does not support our hypothesis. In an additional small control group, RgIA4 was applied in combination with VA ECMO (n = 4; data not shown). Although the number of these experiments was too small to draw firm conclusions, cytokine levels did not seem to differ from VA ECMO alone. Our results might indicate that the conopeptide has no impact on cytokine release in this experimental setting. It remains to be investigated, which nAChR-independent anti-inflammatory mechanisms are induced by AAT in this experimental setting.

Repeated blood analyses in the same animal were performed, which would require statistical tests such as an analysis of variance (ANOVA) for repeated measurements. However, the use of parametric tests on such low n-numbers is questionable, because a normal data distribution cannot be granted. Therefore, we preferred the non-parametric Kruskal–Wallis test followed by pairwise post hoc analysis with the Wilcoxon–Mann–Whitney. To be on the safe side, we additionally performed ANOVA followed by the post hoc Tukey test on the cytokine data, including the factors “group” and “time points”. The analysis regarding the factor “group” revealed significantly elevated concentrations of TNF-α, IL-6 and IL-10 (*p* < 0.001; *p* < 0.001; *p* = 0.001) in response to ECMO compared to sham, and after AAT application, a significant decrease in TNF-α levels (*p* < 0.001) as well as a trend towards lower levels of IL-6 (*p* = 0.08). The difference regarding “group” and IL-6 depended on the “time point”: The concentration of IL-6 after 2 h of ECMO was significantly (*p* < 0.001) elevated compared to sham. Finally, application of AAT led to a significant decrease of IL-6 during ECMO (*p* = 0.05). The results of the multifactorial ANOVA for repeated measurements are in accordance with the results of the non-parametric tests.

This study has several limitations, that altogether suggest that our study should be regarded as a pilot study and that more research is required. (1) ECMO in small animals involves drastic hemodilution. However, the measured concentration of hemoglobin after commencing the ECMO were similar to the values obtained on intensive care units^70^. (2) We used hydroxyethyl starch for priming of the ECMO circuit, because this was done by other authors, who established similar experimental models for ECMO before. In future experiments alternative priming solutions need to be tested, as hydroxyethyl starch is not used anymore due to increased morbidity and mortality in the critically ill^71^. However, since all ECMO groups are treated in the same way, hydroxyethyl starch is probably not responsible for inter-group differences. (3) The application of VA ECMO leads to increased levels of paO_2_, which is known to elevate the concentrations of proinflammatory cytokines^72^. Nevertheless, the levels of paO_2_ were increased in all ECMO groups and no intergroup difference was measured. (4) The duration of VA ECMO in our experiments was 2 h, which only allows investigating the very first phase of VA ECMO-induced inflammation. In patients, ECMO is applied for much longer periods of time, from several days to weeks. (5) Normally, ECMO is performed in critically ill patients, in whom the innate immune system is expected to be pre-activated. In our study, however, we used healthy young adult rats without any pro-inflammatory priming. This might also explain our finding that systemic IL-1β levels did not increase during VA ECMO. It would be of interest, to investigate if the application of a pro-inflammatory stimulus such as lipopolysaccharide before starting experimental VA ECMO induces the release of IL-1β and if this release is inhibited by AAT. Alternatively, due to the short half-life of IL-1β, we might have missed transient increases in circulating IL-1β, which could have been detected by an increased frequency in blood sampling. However, blood sampling is limited due to the relatively small blood volume of the experimental rat, which is also the reason why the number of the parameters tested in this study is limited. (6) It should be mentioned that human AAT was used in the rat and only a single dose was investigated. It was, however, shown before, that human AAT is working in rodents at physiological concentrations^35,45,64,65,73^, but we can never exclude that certain functions of this pleiotropic molecule are species-specific. (7) After unblinding the experimental groups, we became aware that only the animals treated with AAT obtained a single bolus of fentanyl and midazolam after about 1 h of VA ECMO because of acute onset of tachycardia and hypertension, which was interpreted as an acute sign of insufficient sedation under general anesthesia. Because of our blinded experimental design and the legitimate animal welfare requirements, this is an inherent limitation of this kind of exploratory study. Although a single bolus is unlikely to have a strong impact on the cytokine concentrations measured at the end of the experiment, we cannot exclude that the additional medication might be responsible for the anti-inflammatory effects seen in the AAT group. Further studies are needed to clarify a potential impact of AAT on anesthesia. (8) Finally, we did not elucidate the mechanism of action of AAT in our experimental setting.

In conclusion, the circulating levels of TNF-α, IL-6 and IL-10 increase within 2 h of experimental VA ECMO in the rat. The liberation of these cytokines seems to be attenuated by treatment with human AAT, suggesting that AAT might protect against the ECMO-induced systemic inflammation and immune paralysis. More studies are needed to clarify the mechanisms by which AAT inhibits cytokine release in vivo and to develop an experimental setting that more closely mimics the inflammatory situation in critically-ill patients.

## Material and methods

### Animals

All animals received human care in compliance with the guidelines ‘Principles of Laboratory Animal Care’ formulated by the National Society for Medical Research and the ‘Guide for the Care and Use Laboratory Animals’ published by the National Institute of Health (NIH Publication No. 88-23, received 1996). All scientific procedures on living animals were approved by the local committee for animal care of the regional council (No. G 28/2017; Regierungspraesidium Giessen, Germany) in accordance with the German animal welfare law, the ARRIVE guidelines and the European legislation for the protection of animals used for scientific purposes (2010/63/EU). Male Lewis rats (weighing 300 to 350 g) from Janvier Labs (Le Genest St. Isle, France) were housed at 22 °C, 55% relative humidity, and a day/night cycle of 14/10 h, with access to standard chow and water ad libitum.

### Design of the study

Rats were randomly divided into five groups by lot to undergo sham protocol (n = 13), VA ECMO (n = 13), VA ECMO with infusion of AAT (n = 7), VA ECMO with infusion of RgIA4 (n = 4), a specific antagonist of nAChRs containing subgroups α9/α10^46^, or VA ECMO with infusion of AAT and RgIA4 (n = 7). Due to the experimental setup, the investigator was blinded to the different groups, but unblinded to the use of ECMO. Rats of the sham group were cannulated but not connected to ECMO. Hemodynamic measurements were performed during VA ECMO at intervals of 15 min and blood was drawn for cytokine measurement every 30 min. The experiment was finished 2 h after starting VA ECMO and animals were exsanguinated. The cannulation strategy and time course of the study are displayed in Fig. 1.

### Anesthesia and surgery

Anesthesia was induced in Lewis rats by inhalation of isoflurane (5%; Baxter, Unterschleissheim, Germany) in 100% oxygen. Thereafter, rats were intubated endotracheally (16 G cannula, 1.7 mm, B. Braun, Melsungen, Germany) and ventilated (Harvard Inspira, Harvard Apparatus, Cambridge, UK) in a weight-adjusted and volume-controlled manner. The tidal volume and respiratory rate were calculated by the respirator after entering the body weight (tidal volume = 0.0062 × body weight (kg)^^1.01^; respiratory rate = 53.3 × body weight (kg)^^−0.26^). After endotracheal intubation, the concentration of isoflurane was adapted between 1.5 and 2.5%, according to the surgical procedure, cardiac rate and blood pressure. End-tidal CO_2_ was measured continuously (MicroCapStar, CWE, Ardmore, Pennsylvania, USA). A continuous electrocardiogram (ECG) was recorded to monitor anesthesia. Rats were placed on a heating pad, and a rectal temperature probe was inserted to enable the maintenance of a body temperature of 36.5 °C.

**Figure 5:**
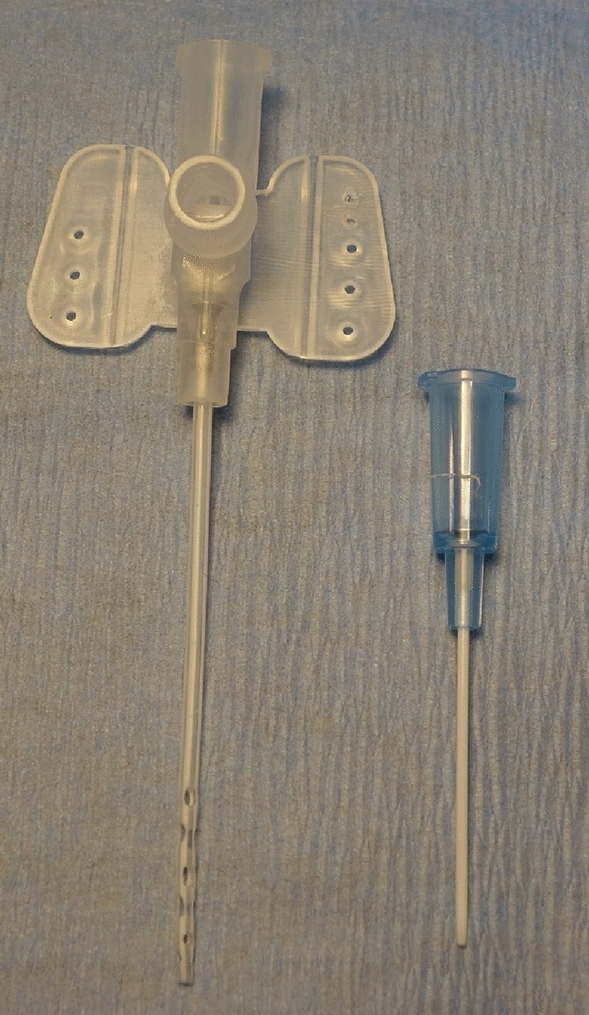
Venous and arterial ECMO cannula. The modified multi-orifice 17 G cannula (1.5 mm, B. Braun, cannula with white connector) was inserted into the internal jugular vein and carefully moved into the right atrium for venous drainage. The oxygenated blood from the ECMO was returned to the femoral artery via a 22 G catheter (0.9 mm, Terumo, cannula with blue connector).

The lateral tail vein was punctured with a 24 G cannula (0.7 mm, B. Braun) for continuous infusion of an isotonic solution (Sterofundin ISO, 10 ml/kg/h; B. Braun), fentanyl (10 μg/kg/h; Albrecht GmbH, Aulendorf, Germany) and midazolam (2 mg/kg/h; Roche, Basel, Switzerland). After cannulation of the tail artery with a 24 G catheter (0.7 mm, B. Braun), the arterial blood pressure was measured continuously. Then, the right femoral artery was cannulated with a 22 G catheter (0.9 mm, Terumo, Eschborn, Germany, Fig. 5) for arterial return from the ECMO. A 2F pressure–volume-catheter (0.7 mm, SPR-838, Millar, Houston, TX, USA) was placed into the left ventricle through the right carotid artery. After intravenous administration of a heparin bolus (400 IU/kg, Ratiopharm, Ulm, Germany), a modified multi-orifice 17 G cannula (1.5 mm, B. Braun, Fig. 5) was inserted into the right jugular vein, and moved carefully to the right atrium for venous drainage to the ECMO. Whenever the rats showed signs of decreased anesthetic effects like tachycardia with hypertension during VA ECMO, a bolus of fentanyl 6 µg/kg bodyweight and midazolam 1.5 mg/kg bodyweight was given. The experimental setting is summarized in Fig. 1A.

### Treatment with AAT and conopeptide RgIA4

All animals of the AAT group received the human AAT preparation Prolastin (Grifols, Frankfurt, Germany), 100 mg/kg bodyweight, infused by a syringe pump (Model 11 Plus, Harvard Apparatus, Cambridge, UK) over 5 min after cannulation of the lateral tail vein. The conopeptide RgIA4 was produced and characterized as described before^45^. RgIA4 was administered after positioning of the venous line as a bolus of 2 nmol and thereafter, added to the continuous infusion at a concentration of 38.8 nmol/kg/h. AAT was infused after the bolus injection of RgIA4 in the subgroup treated with both drugs. Afterwards, the continuous infusion containing RgIA4 was started. Animals of the other groups received sodium chloride 0.9% as a vehicle instead of AAT and/or RgIA4.

### Extracorporeal membrane oxygenation

The uncoated ECMO circuit consisted of silicone and polyvinylchloride tubing, a venous reservoir (M. Humbs, Valley, Germany), a roller-pump (Verderflex Vantage 3000, Castleford, UK) and a membrane oxygenator (M. Humbs) with a three-layer polypropylene hollow fiber membrane (Oxyphan Membrana, Wuppertal, Germany). The circuit was primed with 11 mL 6% hydroxyethyl starch (Voluven, Fresenius Kabi, Bad Homburg, Germany) and 250 IU Heparin (Ratiopharm, Ulm, Germany). After an intravenous sedation bolus of fentanyl 6 µg/kg bodyweight and midazolam 1.5 mg/kg bodyweight for the preemptive treatment of the drug sequestration to the ECMO circuit, the blood flow through the extracorporeal circuit was initiated with a flow rate of 45 mL/kg/min and continuously increased to 90 mL/kg/min. While the oxygen fraction on the membrane was set to 0.5, the gas flow was adjusted between 20 and 35 mL/min to adjust the paCO_2_ between 30 and 40 mmHg. The anesthetic depth was regulated by the concentration of isoflurane, which was adapted according to the heart rate of the rats. Further, midazolam (2 mg/kg body weight/h) and fentanyl (10 µg/kg body weight/h) were administered continuously.

### Hemodynamic measurements

Hemodynamic parameters including CO, SV, LVEDV, left ventricular EF, SAP, DAP, MAP and HR were recorded at 15 min intervals. The left ventricular pressure–volume-catheter was calibrated by parallel conductance with the central venous administration of 50 µL sodium chloride 10% prior to commencing the ECMO.

### Blood analyses

Blood samples (100 µL) were collected at baseline (prior to ECMO) and every hour after commencing ECMO. Blood gases, hemoglobin, pH, bicarbonate, lactate, and electrolytes were measured (ABL800, Radiometer, Copenhagen, Denmark).

### Enzyme-linked immuno-sorbent assay (ELISA)

Blood was drawn (400 µL) at baseline (prior to ECMO) and every 30 min after starting ECMO, centrifuged at 3000 g for 5 min and the plasma was stored at − 80 °C for further analysis. To quantify the inflammatory response, TNF-α, IL-6, IL-1β and IL-10 were measured by ELISA (ELISA Kit, R&D Systems, Wiesbaden, Germany) according to manufacturer’s instructions. Repeated freezing and thawing of the samples was avoided.

### Statistics

All data are given as median with interquartile ranges (25th and 75th percentile). The Kruskal–Wallis test was used for comparison between groups at the same time point. Further, pairwise posthoc analysis was performed with the Wilcoxon–Mann–Whitney test. All statistical analyses were performed using SPSS Version 20 (IBM, Stuttgart, Germany). GraphPad Prism Version 7 was used for data presentation (GraphPad Software, San Diego, CA, USA).

## Supplementary Information

Supplementary Table S1.
