# Supplementary material for: Application of alpha1-antitrypsin in a rat model of veno-arterial extracorporeal membrane oxygenation
Source: Sci Rep. 2021 Aug 4;11:15849. doi: 10.1038/s41598-021-95119-y (PMC8339069; doi:10.1038/s41598-021-95119-y)
Supplement: Supplementary file 1 — Supplementary Table S1. [file 41598_2021_95119_MOESM1_ESM.docx]

**Data Supplement**

**Application of alpha1-antitrypsin in a rat model of veno-arterial extracorporeal membrane oxygenation**

Edinger F.^1^, Schmitt C.^1^, Koch C.^1^, McIntosh J.M.^2,3,4^, Janciauskiene S.^5,7^, Markmann M.^1^, Sander M.^1^, Padberg W.^6^, Grau V.^6,7^

^1^Department of Anesthesiology, Intensive Care Medicine and Pain Therapy, Justus-Liebig University of Giessen, Giessen, Germany

^2^George E. Wahlen Veterans Affairs Medical Center, Salt Lake City, Utah, USA

^3^Department of Biology, University of Utah, Salt Lake City, Utah, USA

^4^Department of Psychiatry, University of Utah, Salt Lake City, Utah, USA

^5^Department of Respiratory Medicine, Hannover Medical School, Hannover, Germany

^6^Laboratory of Experimental Surgery, Department of General and Thoracic Surgery, Justus-Liebig-University of Giessen, Giessen, Germany

^7^Member of the German Centre for Lung Research (DZL)

**Supplementary Table 1:** Blood parameters continued

**Supplementary Table 1:** Blood parameters continued

|  |  |  |  |  |
| --- | --- | --- | --- | --- |
| Parameter | Group | 0 h | 1 h | 2 h |
| pH | Sham | 7.35 (7.32 – 7.38) | 7.35 (7.30 – 7.38) | 7.34 (7.30 – 7.36) |
|  | ECMO | 7.36 (7.32 – 7.39) | 7.46 (7.42 – 7.49)*** | 7.41 (7.31 – 7.44)* |
|  | ECMO + AAT | 7.35 (7.33 – 7.37) | 7.42 (7.34 – 7.43) | 7.42 (7.36 – 7.45) |
|  | ECMO+AAT+RgIA4 | 7.35 (7.32 – 7.40) | 7.46 (7.43 – 7.47) | 7.41 (7.40 – 7.46) |
| Bicarbonate | Sham | 21.1 (20.1 – 24.1) | 21.9 (21.5 – 23.8) | 21.7 (20.8 – 23.2) |
| [mmol/l] | ECMO | 22.2 (20.9 – 23.5) | 24.2 (23.5 – 25.2)** | 22.1 (21.0 – 24.4) |
|  | ECMO + AAT | 21.5 (20.1 – 22.6) | 23.3 (20.7 – 24.5) | 23.8 (22.7 – 26.6) |
|  | ECMO+AAT+RgIA4 | 22.4 (18.3 – 22.7) | 24.2 (23.6 – 24.6) | 23.4 (23.0 – 24.3) |
| BE | Sham | -4.1 (-5.3 – -0.5) | -3.0 (-3.4 – -0.8) | -3.1 (-4.3 – -1.6) |
|  | ECMO | -2.5 (-4.3 – -1.1) | -0.6 (-1.1 – 0.4) | -2.8 (-4.1 – -0.5) |
|  | ECMO + AAT | -3.5 (-5.2 – -2.2) | -1.3 (-4.4 – 0.0) | -0.8 (-2.1 – 2.4) |
|  | ECMO+AAT+RgIA4 | -2.4 (-7.6 – -2.0) | -0.3 (-0.9 – 0.1) | -1.2 (-1.7 – -0.2) |
| Sodium | Sham | 146 (144 – 149) | 144 (143 – 148) | 146 (145 – 152) |
| [mmol/l] | ECMO | 144 (141 – 146) | 143 (142 – 145) | 146 (142 – 147) |
|  | ECMO + AAT | 147 (143 – 147) | 143 (143 – 144) | 145 (143 – 148) |
|  | ECMO+AAT+RgIA4 | 143 (142 – 146) | 142 (142 – 146) | 144 (143 – 147) |
| Potassium | Sham | 3.7 (3.3 – 3.9) | 3.8 (3.3 – 4.5) | 3.8 (3.6 – 4.0) |
| [mmol/l] | ECMO | 3.7 (3.2 – 4.1) | 3.8 (3.6 – 3.9) | 4.0 (3.9 – 4.2) |
|  | ECMO + AAT | 3.3 (3.2 – 3.6) | 3.6 (3.4 – 3.7) | 3.9 (3.6 – 4.4) |
|  | ECMO+AAT+RgIA4 | 3.9 (3.5 – 4.2) | 3.8 (3.2 – 4.0) | 4.3 (3.5 – 4.4) |
| Calcium | Sham | 1.34 (1.31 – 1.42) | 1.35 (1.26 – 1.43) | 1.32 (1.30 – 1.47) |
| [mmol/l] | ECMO | 1.35 (1.31 – 1.42) | 1.28 (1.23 – 1.38) | 1.30 (1.23 – 1.39) |
|  | ECMO + AAT | 1.31 (1.23 – 1.34) | 1.25 (1.23 – 1.28)# | 1.22 (1.19 – 1.25)# |
|  | ECMO+AAT+RgIA4 | 1.40 (1.36 – 1.43) | 1.37 (1.22 – 1.45)§§ | 1.41 (1.24 – 1.44) |
| Chloride | Sham | 114 (111 – 120) | 115 (110 – 119) | 115 (113 – 119) |
| [mmol/l] | ECMO | 111 (110 – 117) | 117 (111 – 119) | 116 (111 – 121) |
|  | ECMO + AAT | 115 (112 – 120) | 119 (119 – 121) | 120 (118 – 121) |
|  | ECMO+AAT+RgIA4 | 116 (112 – 116) | 119 (118 – 119) | 118 (116 – 121) |

Data are presented as median and interquartile ranges (25^th^ and 75^th^ percentile); *, #, *p* ≤ 0.05; **, §§, *p* ≤ 0.01; ***, *p* ≤ 0.001; *, SHAM vs. ECMO; #, ECMO vs. ECMO + AAT; § ECMO + AAT vs. ECMO + AAT + RgIA4; AAT, α-1-antitrypsin; ECMO, extracorporeal membrane oxygenation; BE, base excess.
